# Reproductive factors and specific histological types of breast cancer: prospective study and meta-analysis

**DOI:** 10.1038/sj.bjc.6604853

**Published:** 2009-02-03

**Authors:** G K Reeves, K Pirie, J Green, D Bull, V Beral

**Affiliations:** 1 Cancer Epidemiology Unit, Nuffield Department of Clinical Medicine, University of Oxford, Oxford, UK

**Keywords:** breast cancer histology, menarche, age at first birth, parity, menopause

## Abstract

Little is known about how reproductive factors affect the risk of breast cancers of different histology. In an analysis of prospective data on 1.2 million middle-aged UK women, we used proportional hazards models to estimate the relative risks of six histological types in relation to menarche, childbearing and menopause. During 8.7 million person-years of follow-up, 17 923 ductal, 3332 lobular, 1062 tubular, 944 mixed ductal lobular, 330 mucinous and 117 medullary cancers were diagnosed. The effect of both age at menarche and age at first birth was greatest for lobular tumours; relative risks per 5-year increase in age at menarche for ductal, lobular, and tubular cancers were 0.93 (0.87–0.99), 0.65 (0.56–0.76), and 0.75 (0.57–0.98), respectively (*P*-value for heterogeneity=0.0001); and the relative risks per 5-year increase in age at first birth were 1.10 (1.07–1.12), 1.23 (1.17–1.29), and 1.13 (1.03–1.23), respectively (*P*-value for heterogeneity=0.0006). Increasing parity reduced the risk of each tumour type, except medullary cancers, but the reduction in risk was greater for mucinous cancers than for any other subtype considered (*P*<0.05 for comparison with each other subtype in turn). The effect of menopause did not vary significantly by tumour histology. Meta-analysis of published results on the effects of age at menarche and age at first birth on ductal and lobular cancers were in keeping with our findings.

Despite the fact that reproductive factors are among the most established risk factors for breast cancer, little is known about whether their effects differ by tumour histology. Few studies of breast cancer in relation to reproductive factors have had sufficient power to estimate reliably the risk of specific subtypes according to detailed reproductive history. We report here on the relationship between age at menarche, parity, age at first birth and age at menopause, and the risk of six histological subtypes of breast cancer in a large prospective study of women in the UK, and present meta-analyses of published results.

## Materials and methods

The Million Women Study recruited 1.3 million middle-aged women in 1996–2001, who completed a questionnaire about reproductive factors, sociodemographic factors and other personal characteristics. They were resurveyed about 3 years after recruitment to update information on menopausal status and other factors, with a 65% response rate. Full details of the study design and methods are described elsewhere (The Million Women Study Collaborative Group, 1999) and questionnaires can be viewed at http://www.millionwomenstudy.org. All participants were flagged on the National Health Service (NHS) Central Registers so that cancer registrations and deaths are routinely notified to the study investigators. These registers provide information on the date of each such event and the cancer site and morphology codes using the 10th revision of the International Classification of Diseases (ICD10) (World Health Organisation, 1992). All participants gave their written consent and ethical approval was granted by the Oxford and Anglia Multi-Centre Research Ethics Committee.

The relationship between the risk of incident invasive breast cancer and various reproductive factors was examined separately for six histological subtypes: ductal (ICD10-O code 8500/3), lobular (ICD10-O code 8520/3), mixed ductal lobular (ICD10-O code 8522/3), tubular (ICD10-O code 8211/3) medullary (ICD10-O code 8510/3), and mucinous (ICD10-O code 8480/3). The variables for these analyses were derived from the information provided at baseline, except for those related to menopause, and to hormone-replacement therapy (HRT) use, which were updated from the resurvey, wherever possible. At each point of contact, a woman's menopausal status and HRT use was defined according to the criteria outlined previously (Million Women Study Collaborators, 2003). For all women who reported being pre- or perimenopausal at last contact, menopausal status was treated as unknown beyond 4 years after last contact. Because HRT use is known to be strongly related to age at menopause, analyses of menopausal status and age at menopause were restricted to never-users of HRT. Women diagnosed with any invasive cancer other than non-melanoma skin cancer (ICD10 C44) before recruitment were excluded from all analyses. For the remaining women, person-years were contributed from the date of recruitment until the date of registration for a breast cancer of interest, the date of death, or the end of follow-up, which was defined as 31 December 2006 for all regions, except for the Thames and West Midlands (30 June 2006), North and Yorkshire and Mersey (31 December 2005) and Scotland (31 December 1999). Women diagnosed with any non-breast (or non-melanoma skin) cancer during follow-up were censored at the diagnosis date.

Proportional hazards models were used to estimate the relative risk (RR) of developing specific histological types of breast cancer according to age at menarche, parity, age at first birth, menopausal status, and age at menopause with attained age as the underlying time variable. All analyses were routinely stratified by broad geographical region (10 regions corresponding to the 10 areas covered by the cancer registries providing data), and adjusted for quintiles of socioeconomic group based on deprivation index (Townsend *et al*, 1988), use of HRT (never, past, current use of duration: <5 years, 5–9 years, 10+ years), body mass index in kg/m^2^ (<25, 25–29, 30+), family history of breast cancer (no, yes), average alcohol consumption (0, 1–2, 3–6, 7–14, 15+ drinks/week), and where appropriate, for age at menarche (<12, 12–13, 14+), parity (0, 1, 2, 3, 4+), age at first birth (nulliparous, <20, 20–24, 25–29, 30+) and time since menopause (premenopausal, perimenopausal, postmenopausal with time since menopause: <5, 5–9, 10+ years). Time since menopause and HRT use were treated as categorical time-dependent variables. Women with missing values for a given adjustment variable were assigned to a separate category for that variable. To assess the effect of including women with missing information for some adjustment factors, analyses were repeated for those with known values for all adjustment variables.

Where analyses involve risk comparison across more than two categories, variances were estimated by treating the RRs as floating absolute risks (Easton *et al*, 1991) and results are, therefore, presented in the form of relative risks and their corresponding floated confidence intervals. Results in the text, which refer to a specific comparison of two categories, or to an estimate of trend, are presented in the form of conventional RRs and their corresponding confidence intervals. All analyses were performed using the statistical package Stata, version 9.2 (StataCorp, 2007). Formal tests of heterogeneity in the relationship between various reproductive factors and breast cancer risk by histological type were carried out using a competing risks approach. Although results are presented for all six subtypes of breast cancer considered, tests of heterogeneity were sometimes restricted to ductal, lobular and tubular cancers as mixed ductal lobular cancers cannot be assumed to be distinct from ductal or lobular tumours, and the numbers of medullary and mucinous cancers were usually too few to allow reliable comparisons with the other subtypes.

We also conducted a meta-analysis of published data for ductal and lobular cancers based on relevant studies identified by 31 August 2008 through searches of PUBMED, and supplemented by searches of reference lists in identified papers. Summary relative risks were estimated by calculating the weighted average of the study-specific log RRs, with weights proportional to the inverse of the variances of the study-specific log relative risks. Studies were grouped according to study design, and summary estimates were calculated separately for case–control and cohort studies.

## Results

A total of 1 193 604 women, aged 50–64 years, without previous cancer (except non-melanoma skin cancer) were followed up for incident breast cancer over 8.7 million person-years. During this period, 27 397 were registered with breast cancer with a median age at diagnosis of 60 years (inter-quartile range: 56–64). The three commonest tumour types were ductal (65% of the total), lobular (12%) and tubular (4%) (Table 1). Mucinous and medullary tumours were rare, comprising 1 and 0.4%, respectively, of all breast cancers. Mixed ductal lobular tumours comprised 3% of the total. The median age at diagnosis (and inter-quartile range) among women diagnosed with ductal, lobular, tubular, mixed ductal lobular, medullary, and mucinous tumours was 59 (56–63), 60 (56–64), 58 (55–62), 60 (56–64), 59 (55–63) and 61 (58–65), respectively. Table 1 also presents RRs for each of the six types considered, together with the corresponding numbers of cases, according to categories of age at menarche, parity, age at first birth, menopausal status and age at menopause.

**Figure 1:**
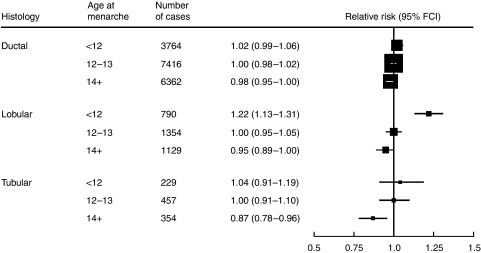
Relative risk of specific histological types of breast cancer according to age at menarche.

The RR of each of the three commonest types of breast cancer, that is ductal, lobular and tubular tumours, decreased with increasing age at menarche. However, the magnitude of the trend in RR per 5-year increase in age at menarche varied considerably by type (*χ*_2_^2^=17.8; *P*=0.0001; Figure 1); the largest reduction in risk was observed for lobular (0.65, 0.56–0.76), and the smallest for ductal cancer (0.93, 0.87–0.99). By contrast, there was little evidence of a decrease in the risk of medullary or mucinous cancers with increasing age at menarche (corresponding RRs=2.03 and 1.39, respectively), although the confidence intervals were wide (Table 1).

There was a significant decrease in risk with increasing parity for every subtype of breast cancer examined except medullary cancer (Table 1). For ductal, lobular and tubular cancers, there was no heterogeneity in the magnitude of this trend (*χ*_2_^2^=3.9; *P*=0.1). The decrease in the risk of mixed ductal lobular cancers (RR per birth=0.89, 0.83–0.95) was similar to that for ductal (0.89, 0.88–0.91) and lobular cancer (0.92, 0.89–0.96). There was, however, a greater decrease in risk with increasing parity for mucinous cancer (0.78, 0.70–0.87), than for the other types (*P*<0.05 for comparison with each other subtype in turn). In contrast to the decrease in risk with increasing parity seen for five of the tumour subtypes, there was a significant increase in the risk of medullary cancer (1.27, 1.02–1.57) (*P*<=0.005 for comparison with each other subtype in turn; Table 1). The association between parity and breast cancer risk was also estimated among parous women only, with adjustment for age at first birth. The corresponding patterns of risk across histological subtypes were broadly similar to those calculated among all women.

**Figure 2:**
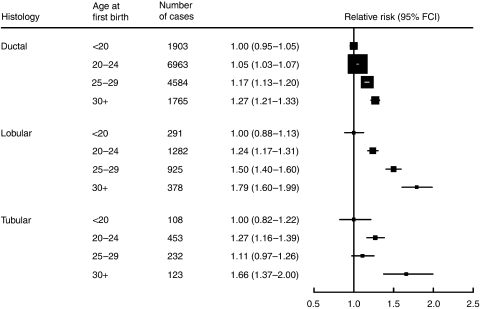
Relative risk of specific histological types of breast cancer according to age at first birth in parous women.

Every histological subtype of breast cancer examined, except medullary tumours, showed an increased risk with increasing age at first birth among parous women. The RRs by age at first birth for ductal, lobular and tubular cancer, are summarised in Figure 2. Although the risk increased with increasing age at first birth for each of the three commonest subtypes, there was significant heterogeneity in the magnitude of the increase (*χ*_2_^2^=15.0; *P*=0.0006). The increase in risk associated with each 5-year delay in age at first birth was greatest for lobular cancer (RR=1.23, 1.17–1.29).

Around 50% (601 568) of women included in the main analyses contributed to the analyses of the effect of menopause among never-users of HRT. Among such women, the relative risk in postmenopausal compared with premenopausal or perimenopausal women was consistently lower than unity for each of the three main types of breast cancer (Table 1), Although the reduction in risk among postmenopausal women was less marked for ductal cancers compared with lobular and tubular cancers, this variation across the three main subtypes was not significant (*χ*_2_^2^=5.1; *P*=0.08). Nor was there any significant difference, among postmenopausal never-users of HRT, in the trend in risk associated with a 5-year increase in age at menopause across the three commonest subtypes (*χ*_2_^2^=1.7; *P*=0.4), although once again the trend was least marked for ductal cancers. There were too few cases of mucinous and medullary breast cancers among pre- and perimenopausal women to reliably assess their relationship with menopausal status but among postmenopausal never users of HRT, increasing age at menopause was associated with a non-significant trend of decreasing risk for mucinous cancer (RR=0.73, 0.51–1.05), and of increasing risk for medullary cancer (RR=1.38, 0.79–2.40).

**Figure 3:**
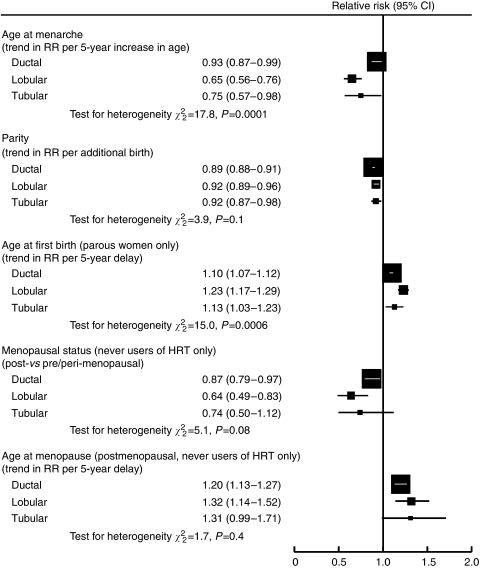
Summary of relationships between reproductive factors and specific histological types of breast cancer.

The relationships between the various reproductive factors and the three most common histological types of breast cancer considered here are summarised in Figure 3. Of all the risk factors considered, only age at menarche and age at first birth showed a clear difference in effect across these three subtypes; both factors being most strongly related to lobular cancers. The RR estimates shown in Figure 3 were not materially altered when analyses were restricted to women with valid information on all adjustment variables.

**Figure 4:**
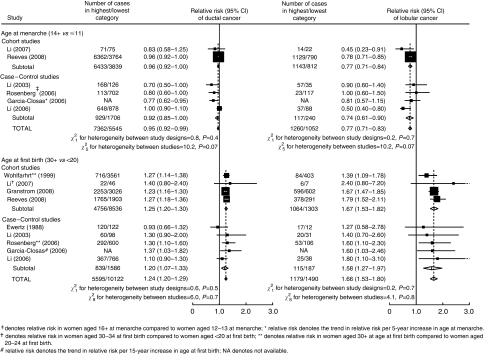
Meta-analysis of published results on the relation between age at menarche and age at first birth, and the risk of ductal and lobular breast cancer.

A meta-analysis of previous studies that have assessed the effect of age at menarche and/or age at first birth on the risk of both ductal and lobular breast cancer, together with the findings presented here, is shown in Figure 4. Corresponding results for other histological subtypes were too sparse to merit meta-analysis. Because of differences in the way in which the relationships were assessed in each study, it was not possible to derive a comparable estimate of relative risk from all of the previous studies. In particular, findings from three studies (Rosen *et al*, 1982; Stalsberg *et al*, 1989; Claus *et al*, 1993) could not be included in the meta-analysis because they were based on analyses conducted only within breast cancer cases, and findings on age at first birth from another study (LiVoisi *et al*, 1982) could not be incorporated because they were based on a different reference group. None of the five previous studies (Li *et al*, 2003, 2006, 2007; Garcia-Closas *et al*, 2006; Rosenberg *et al*, 2006) for which comparable estimates of the effect of age at menarche were available, reported a significant difference in the effect of age at menarche on lobular compared with ductal cancer, although one study did find a significantly greater effect of age at menarche on lobular compared with ductal cancer, specifically among postmenopausal women (Li *et al*, 2008). The overall relative risk in women whose age at menarche was 14 or more, compared to less than 12, was 0.95 (0.92–0.99) for ductal cancer and 0.77 (0.71–0.83) for lobular cancer. All eight previous studies (Ewertz and Duffy, 1988; Wohlfahrt *et al*, 1999; Li *et al*, 2003, 2006, 2007; Garcia-Closas *et al*, 2006; Rosenberg *et al*, 2006; Granstrom *et al*, 2008) included in the meta-analysis of the effect of age at first birth showed a greater effect on lobular than ductal cancer; however, most of these studies had limited power and only one (Wohlfahrt *et al*, 1999) reported a significant difference in the RRs for lobular and ductal cancers according to age at first birth. Overall, the RR in women whose age at first birth was 30 or more, as opposed to less than 20, was 1.24 (1.20–1.29) for ductal cancer and 1.66 (1.53–1.80) for lobular cancer. In analyses of age both at menarche and at first birth, there was no evidence of any material variability in the RRs of ductal and lobular cancer according to study design, or between studies.

## Discussion

In this large prospective study including 27 397 women with incident invasive breast cancer, we have shown that certain aspects of reproductive history are associated with different risks of developing different histological types of breast cancer. In particular, the well-known effects of age at menarche and of age at first birth vary significantly across tumour type, with the greatest effects seen for lobular breast cancer. Increasing parity was associated with reductions in risk for ductal, lobular, tubular and mucinous cancers, with a substantially greater effect for mucinous cancers than for the other tumour types. By contrast, the risk of medullary cancer increased with increasing parity. For most of the reproductive factors considered, the relative risks for mixed ductal lobular cancer were intermediate between those found for ductal and lobular cancer.

Several studies (LiVoisi *et al*, 1982; Rosen *et al*, 1982; Ewertz and Duffy, 1988; Stalsberg *et al*, 1989; Claus *et al*, 1993; Wohlfahrt *et al*, 1999; Li *et al*, 2003, 2006, 2007, 2008; Garcia-Closas *et al*, 2006; Rosenberg *et al*, 2006; Granstrom *et al*, 2008) have published findings on the relationship between reproductive factors and specific histological types of breast cancer and, although the majority found no significant differences in the effects of reproductive risk factors according to histological type, most lacked statistical power. Indeed only three studies (Stalsberg *et al*, 1989; Wohlfahrt *et al*, 1999; Li *et al*, 2008) have reported significant differences according to histological type for at least one aspect of reproductive history; two (Stalsberg *et al*, 1989; Wohlfahrt *et al*, 1999) found that increasing age at first birth was associated with a significantly greater relative risk of lobular cancer and/or tubular cancer than of ductal cancer, and one (Li *et al*, 2008) found a significantly greater effect of age at menarche on lobular compared with ductal breast cancer among postmenopausal women. A large record-linkage study in Sweden (Granstrom *et al*, 2008) also showed a greater effect of increasing age at first birth for lobular compared with other types, and a smaller effect of low parity on lobular compared with ductal and tubular subtypes; however, there was limited information on potential confounders and no formal tests of heterogeneity by histological subtype were given.

Although few individual studies have had sufficient power to examine reliably the relationship between reproductive history and histology, a meta-analysis of published data on the effect of age at menarche and age at first birth on ductal and lobular cancer, together with the present results, provides strong evidence of material differences in their effects on these two tumour types (Figure 4).

Only five studies have reported on the relationship between reproductive history and the risk of relatively rare subtypes of breast cancer (Rosen *et al*, 1982; Stalsberg *et al*, 1989; Claus *et al*, 1993; Wohlfahrt *et al*, 1999; Li *et al*, 2006). For mucinous tumours (1% of all breast cancers in our cohort), two previous studies (Stalsberg *et al*, 1989; Wohlfahrt *et al*, 1999) found that the risk in parous compared with nulliparous women was significantly lower than for ductal cancers. Our results are consistent with those reports as we found a significantly greater protective effect per birth for mucinous cancer (RR per additional birth=0.78, 0.70–0.87) than for every other subtype examined.

For medullary tumours (0.4% of the total), our findings suggest that several indices of reproductive history may have a qualitatively different relationship with risk compared with other breast cancer subtypes. For example, medullary cancer risk increased with increasing age at menarche and with increasing parity but decreased with increasing age at first birth – the opposite of what is found for the more common subtypes. The most notable difference in the effect of parity on medullary compared with all other subtypes examined here was statistically significant and as far as we are aware, no previous study has reported such a difference.

The mechanism by which reproductive factors influence the development of breast cancer is not yet well understood, although it is widely believed that the protective effects of a birth may be due to the hormonal changes that occur during pregnancy and lactation (Thomas, 1984). HRT results in a greater increase in the incidence of lobular and tubular than of ductal cancers (Reeves *et al*, 2006), and lobular breast tissue may be more responsive than ductal to hormones involved in reproduction (Stalsberg *et al*, 1989). Alternatively, as lobular cancers are more likely than other subtypes to be hormone sensitive (Li *et al*, 2005) the findings may reflect a greater effect of certain reproductive factors on hormone-sensitive breast cancers (Althuis *et al*, 2004). Although our findings show clearly that age at first birth has a stronger effect on lobular than ductal cancer, they do not suggest a corresponding difference in the effect of parity on these two subtypes.

The Million Women Study is well placed to investigate the relationships between reproductive history and specific histological subtypes of breast cancer as its large size affords sufficient power for reliable comparisons. Complete follow-up for incident cancers and information on tumour histology is coded and routinely provided to the investigators by the NHS Central Registers. Such information has been demonstrated to be sufficiently valid to be used in epidemiological studies (Gathani *et al*, 2005). Although age at menarche, parity and age at first birth, and most other adjustment variables, were reported at baseline, those variables which may be subject to change, such as menopausal status and HRT use, have been updated using information obtained at resurvey for the majority of women. It is, of course, still possible that some women may have been misclassified, but this would not be expected to give rise to spurious differences in relationships according to tumour histology. Although this is the largest study to date of the effect of reproductive factors on breast cancer histology, our findings for the rarer tumour types, mucinous and medullary cancers, are based on comparatively small numbers.

## Figures and Tables

**Table 1:** Relative risk^a^ of specific histological types of breast cancer according to reproductive history

	**Ductal**		**Lobular**		**Tubular**		**Mixed ductal lobular**		**Mucinous**		**Medullary**	
	**Cases**	**RR (95% FCI)**	**Cases**	**RR (95% FCI)**	**Cases**	**RR (95% FCI)**	**Cases**	**RR (95% FCI)**	**Cases**	**RR (95% FCI)**	**Cases**	**RR (95% FCI)**
*Age at menarche*												
<12	3764	1.02 (0.99–1.06)	790	1.22 (1.13–1.31)	229	1.04 (0.91–1.19)	213	1.13 (0.99–1.30)	59	0.85 (0.66–1.10)	20	0.84 (0.54–1.31)
12–13	7416	1.00 (0.98–1.02)	1354	1.00 (0.95–1.05)	457	1.00 (0.91–1.10)	384	1.00 (0.91–1.10)	136	1.00 (0.85–1.18)	45	1.00 (0.75–1.34)
14+	6362	0.98 (0.95–1.00)	1129	0.95 (0.89–1.00)	354	0.87 (0.78–0.96)	319	0.94 (0.84–1.05)	128	1.04 (0.87–1.24)	50	1.27 (0.96–1.69)
Trend (per 5 years)		0.93 (0.87–0.99)		0.65 (0.56–0.76)		0.75 (0.57–0.98)		0.74 (0.55–0.99)		1.39 (0.83–2.33)		2.03 (0.83–4.96)
												
*No. of livebirths* b												
0	2334	1.00 (0.96–1.04)	383	1.00 (0.90–1.11)	131	1.00 (0.84–1.19)	121	1.00 (0.84–1.20)	56	1.00 (0.77–1.30)	10	1.00 (0.54–1.86)
1	2773	0.89 (0.86–0.92)	512	1.02 (0.94–1.11)	173	0.99 (0.85–1.15)	149	0.93 (0.79–1.09)	48	0.63 (0.47–0.83)	12	0.91 (0.51–1.59)
2	7531	0.80 (0.78–0.82)	1465	0.95 (0.90–1.00)	444	0.83 (0.76–0.91)	401	0.82 (0.74–0.91)	144	0.66 (0.56–0.78)	40	0.98 (0.72–1.35)
3+	5256	0.71 (0.69–0.73)	963	0.81 (0.76–0.86)	314	0.81 (0.72–0.91)	271	0.71 (0.63–0.80)	82	0.43 (0.34–0.53)	54	1.65 (1.25–2.17)
Trend (per birth)		0.89 (0.88–0.91)		0.92 (0.89–0.96)		0.92 (0.87–0.98)		0.89 (0.83–0.95)		0.78 (0.70–0.87)		1.27 (1.02–1.57)
												
*Age at first birth* c												
<20	1903	1.00 (0.95–1.05)	291	1.00 (0.88–1.13)	108	1.00 (0.82–1.22)	86	1.00 (0.80–1.25)	34	1.00 (0.70–1.44)	22	1.00 (0.62–1.60)
20–24	6963	1.05 (1.03–1.07)	1282	1.24 (1.17–1.31)	453	1.27 (1.16–1.39)	373	1.24 (1.13–1.37)	121	0.94 (0.79–1.12)	50	0.73 (0.56–0.96)
25–29	4584	1.17 (1.13–1.20)	925	1.50 (1.40–1.60)	232	1.11 (0.97–1.26)	240	1.37 (1.21–1.56)	86	1.11 (0.90–1.38)	26	0.72 (0.49–1.06)
30+	1765	1.27 (1.21–1.33)	378	1.79 (1.60–1.99)	123	1.66 (1.37–2.00)	103	1.71 (1.39–2.10)	25	0.93 (0.62–1.40)	7	0.59 (0.27–1.28)
Trend (per 5 years)		1.10 (1.07–1.12)		1.23 (1.17–1.29)		1.13 (1.03–1.23)		1.19 (1.08–1.31)		1.02 (0.86–1.22)		0.84 (0.63–1.14)
												
*Menopausal status* d ^,^ e												
Pre/peri	923	1.00	176	1.00	70	1.00	38	1.00	9	1.00	6	1.00
Post	2863	0.87 (0.79–0.97)	460	0.64 (0.49–0.83)	135	0.74 (0.50–1.12)	120	0.95 (0.59–1.54)	53	0.94 (0.34–2.58)	31	1.96 (0.66–5.81)
												
*Age at menopause* f												
<45	252	0.75 (0.66–0.85)	28	0.49 (0.34–0.71)	8	0.41 (0.21–0.83)	10	0.74 (0.40–1.38)	10	2.07 (1.11–3.88)	1	0.21 (0.03–1.49)
45–49	764	0.87 (0.81–0.94)	116	0.78 (0.64–0.94)	39	0.70 (0.50–0.98)	38	1.05 (0.76–1.46)	16	1.39 (0.85–2.28)	10	0.80 (0.42–1.55)
50–54	1451	1.00 (0.95–1.05)	253	1.00 (0.88–1.13)	78	1.00 (0.79–1.26)	60	1.00 (0.77–1.29)	22	1.00 (0.66–1.52)	18	1.00 (0.63–1.59)
55+	310	1.22 (1.09–1.37)	50	1.04 (0.78–1.38)	9	0.71 (0.36–1.38)	12	1.24 (0.70–2.23)	5	1.14 (0.47–2.76)	2	0.61 (0.15–2.51)
Trend (per 5 years)		1.20 (1.13–1.27)		1.32 (1.14–1.52)		1.31 (0.99–1.71)		1.16 (0.88–1.53)		0.73 (0.51–1.05)		1.38 (0.79–2.40)

a Adjusted for age, geographical region, socio-economic status, smoking status, alcohol intake, physical activity, all other indices of reproductive history and HRT use.

b Not adjusted for age at first birth.

c Restricted to parous women only.

d Restricted to never HRT users.

e Confidence intervals denote conventional rather than floated confidence intervals.

f Restricted to postmenopausal never HRT users.
